# Beta-Cell Adaptation to Pregnancy – Role of Calcium Dynamics

**DOI:** 10.3389/fendo.2022.853876

**Published:** 2022-03-25

**Authors:** Marle Pretorius, Carol Huang

**Affiliations:** Department of Biochemistry and Molecular Biology, Cumming School of Medicine, University of Calgary, Calgary, AB, Canada

**Keywords:** pregnancy, beta-cell, calcium, insulin secretion, apoptosis, islets, endoplasmic reticulum

## Abstract

During pregnancy, the mother develops insulin resistance to shunt nutrients to the growing fetus. As a result, the maternal islets of Langerhans undergo several changes to increase insulin secretion in order to maintain glucose homeostasis and prevent the development of gestational diabetes. These changes include an increase in β-cell proliferation and β-cell mass, upregulation of insulin synthesis and insulin content, enhanced cell-to-cell communication, and a lowering of the glucose threshold for insulin secretion, all of which resulting in an increase in glucose-stimulated insulin secretion. Emerging data suggests that a change in intracellular calcium dynamics occurs in the β-cell during pregnancy as part of the adaptive process. Influx of calcium into β-cells is crucial in the regulation of glucose-stimulated insulin secretion. Calcium fluxes into and out of the cytosol, endoplasmic reticulum, and mitochondria are also important in controlling β-cell function and survival. Here, we review calcium dynamics in islets in response to pregnancy-induced changes in hormones and signaling molecules, and how these changes may enhance insulin secretion to stave off gestational diabetes.

## Introduction

It has long been known that during pregnancy, the maternal insulin demand increases due to the physiologic increase in insulin resistance (1). To accommodate this increased demand, pancreatic islets adapt through several mechanisms including increasing insulin synthesis and lowering the threshold for glucose-stimulated insulin secretion (GSIS) (2, 3), which has been demonstrated in both rodents and humans (4–10). Change in calcium dynamics within pancreatic islets, and specifically in various subcellular compartments of the pancreatic β-cell, can affect β-cell function such as insulin secretion and β-cell survival (11–13). This review will explore and summarize the current knowledge on calcium dynamics in pancreatic islets during pregnancy, and the implications in gestational diabetes mellitus (GDM).

## Changes in Islets During Pregnancy

During pregnancy, the maternal pancreatic islets are placed under high demand for insulin production due to an increase in insulin resistance of maternal tissues (8, 14), a physiologic change that encourages the diversion of nutrient from the mother to the developing fetus. In order to accommodate for this increase in insulin demand, studies in rodent islets and β-cell lines have identified several mechanisms involved, including an increase in β-cell proliferation, β-cell size (7, 10, 15), insulin gene expression, insulin synthesis, and insulin content, as well as lowering the glucose threshold for insulin secretion (16, 17). An increase in β-cell mass and number has also been demonstrated in human pregnancy (9, 18). An increase in gap-junction coupling and islet vasculature density are also part of the adaptive mechanism (19, 20). In contrast, there is no significant change in α-cell number or size during pregnancy, and no significant change in the spatial organization of the islets, i.e. the majority of the β-cells form the core of the islet surrounded by α-cells in the periphery (15, 21). Gene ontology analysis of the islet transcriptome during pregnancy has identified enrichment of genes that regulate cell proliferation, apoptosis, response to stress, cell communication, cellular physiological processes such as proteolysis and vesicle trafficking, as well as cellular metabolic processes such as lipid metabolism and electron transport (22–25). Many of these adaptive responses are regulated by pregnancy hormones such as lactogens (15, 26), growth hormone (27–29), estrogen (30, 31), progesterone (20, 32), and other factors such as hepatic growth factors (33)and serotonin (24). Whether these or other pregnancy-associated factors regulate β-cell Ca^2+^ handling directly and contribute to the enhanced insulin secretion observed during pregnancy requires much more investigation, although indirect evidence suggests that they may participate in the regulation of Ca^2+^ dynamics in β-cells.

Estrogen (17 β-oestradiol or E2) level increases throughout pregnancy (34). It acts through the classic nuclear hormone estrogen receptor α and β isoforms (ERα and ERβ) as well as through the G protein-coupled estrogen receptor 1 (GPER1, or GPR30) in both rodents and humans islets (35). The 3 receptors have distinctive functions in β-cells. Activation of ERα regulates insulin synthesis and β-cell survival (31, 36). Activation of ERβ in mouse islets stimulates guanylyl cyclase A and rapidly increases cyclic GMP levels, leading to a reduction in K_ATP_ channel activity in the plasma membrane, an increase in calcium oscillation and cytosolic calcium ([Ca^2+^]_c_), and augments GSIS (37, 38). Mice treated with an ERβ agonist also demonstrated a higher β-cell mass and β-cell proliferation (38). GPER1 (aka GPR30) is a plasma membrane receptor that upon 17 β-estradiol binding, stimulates cGMP synthesis and activates protein kinase G (PKG), leading to closure of the K_ATP_ channel and increases frequency of Ca^2+^ oscillation and intracellular Ca^2+^ concentration, enhancing GSIS (35, 39–42). Martensson et al. found that in GPR30^-/-^ mice, there is a defect in 1^st^-phase insulin secretion *in vivo* and when these islets were tested *in vitro*, E2-stimulated insulin secretion was completely abolished, suggesting its dominant role in regulating E2-mediated insulin secretion (42). GPER1 activation also protects β-cells against apoptosis (43). During pregnancy, estrogen receptor ERα and GPER expression are up regulated in rodent islets (44, 45). Recently, Ma et al. reported activation of the transient receptor potential ankyrin-repeat 1 (TRPA1) channels in INS-1 cells as well as rodent and human β-cells by estradiol metabolites (46). TRPA1 is a cation channel that is activated by a wide variety of exogenous irritants and inflammatory cytokines (47). It has been shown to regulate insulin secretion (48). In this study (46), estradiol metabolites (but not estradiol) induced strong inward current and a robust and sustained elevation in [Ca^2+^]_c_ an increase that closely parallels their effect to enhance GSIS.

Serotonin has been identified as a key regulator of β-cell proliferation during pregnancy. Expression of serotonin, as well as tryptophan hydroxylase-1, the enzyme responsible for serotonin synthesis, was found to increase significantly in rodent islets during pregnancy (24). Inhibition of serotonin synthesis during pregnancy blocks β-cell proliferation, resulting in glucose intolerance. This was found to be downstream of lactogen and prolactin receptor (PRLR) signaling. In human islets, serotonin is secreted by β-cells and it exerts paracrine action on α-cells, inhibiting glucagon secretion (49). Activation of the serotonin receptor, 5-HT2B by a-methyl serotonin maleate salt has been shown to alter [Ca^2+^]_c_ oscillation, causing an increase in both peak duration and distance between peaks in mouse islets, and an increase in insulin secretion from both human and mouse islets (50). Interestingly, exposure of β-cells in culture (MIN6 mouse insulinoma cells) to a selective serotonin reuptake inhibitor (SSRI) reduced ER calcium stores and inhibited ER calcium release and store-operated calcium entry activation (51). SSRIs can also inhibit insulin secretion by inhibiting mitochondrial complex I and II, decrease oxidative respiration, ATP generation and K_ATP_ channel activity, although this study did not measure [Ca^2+^]_c_ (52). Whether the increase in endogenous serotonin in islets during pregnancy regulates intracellular Ca^2+^ dynamics and contributes to GSIS require further investigation.

The corticotropin-releasing hormone (CRH) family of peptides activates the cAMP/PKA signaling pathways, potentiate Ca^2+^ influx through the L-type Ca^2+^ channels and modulate insulin secretion in rat islets (53). CRH and its paralogs, urocortin 1 (Ucn1), Ucn2, and Ucn3 act through their cognate G-protein-coupled receptors, CRH receptor 1 (CRHR1) and CRH receptor 2 (CRHR2); both are expressed in pancreatic β-cells, such as the MIN6 mouse insulinoma cells and primary rodent islets (53–56). A recent study by Simpson et al. found that during mouse pregnancy, urocortin 2 (Ucn2) is up regulated and it acts through CRHR2 to regulate glucose homeostasis, most likely *via* its effect on insulin secretion (57), This conclusion was based on the observation that CRHR2 blockade had no effect on insulin sensitivity or β-cell proliferation while both *in vitro* and *in vivo* blockage of CRHR2 have been shown to attenuate GSIS in mice (58).

Hepatic growth factor (HGF) is another hormone that has been shown to be important in the regulation of β-cell adaptation to pregnancy (33). During pregnancy, there is an increase in HGF level in the serum and HGF expression in the β-cells. Transgenic mice with β-cell specific HGF overexpression had increased glucokinase expression, glucose transport, and insulin secretion (59). In kidney epithelial cells, HGF has been found to inhibit Ca^2+^ release from the ER while in hepatocytes, HGF activates the inositol-triphosphate-PLCγ pathway and causes a rapid rise in [Ca^2+^]_c_ (60). Whether similar response to HGF occurs in β-cells has yet to be determined.

Lastly, many of the changes observed in the islets during pregnancy are due to actions of prolactin and placental lactogens, both signaling through the Prolactin Receptor (PRLR) (15, 26). Work by us and others have shown that PRLR deletion led to impaired glucose tolerance during mouse pregnancy, mainly by dampening pregnancy-induced β-cell proliferation. This results in a smaller β-cell mass, lower serum insulin levels, and reduced pancreatic insulin content. Interestingly, several transcriptome analyses have identified Leucine Rich Repeat Containing 55 (Lrrc55) (61), an auxiliary protein of big-potassium channels (62), as one of the most highly upregulated genes downstream of PRLR in the pancreatic islets during pregnancy (24, 63), and we found that Lrrc55 is a novel, pro-survival factor in β-cells, potentially by regulating calcium handling (25).

## Calcium Dynamics in β-Cells

**Figure 1:**
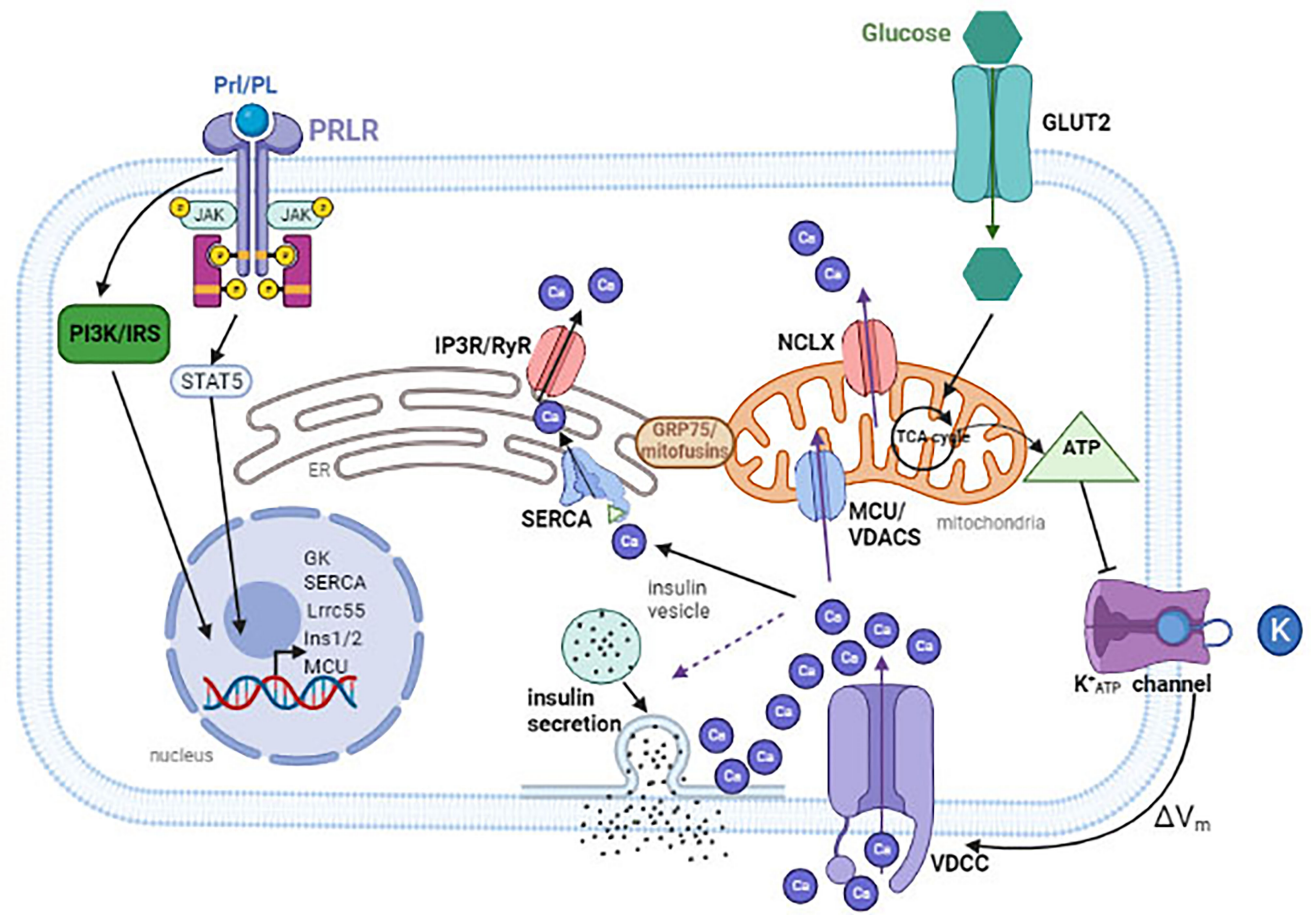
Glucose is transported into the cytoplasm through glucose transporters (GLUT2 in rodents). Once inside, glucose are metabolized in the mitochondria to generate ATP. An increase in ATP to ADP ratio will cause the closure of ATP-sensitive potassium channel (K_ATP_), resulting in membrane depolarization. This leads to the opening of the L-type voltage-dependent Ca^2+^ channels (VDCC) and an influx of Ca^2+^. To maintain [Ca^2+^]_m_, Ca^2+^ enters through the MCU or the VDACs and escapes through the NCLX. Ca^2+^ is pumped into the ER through ATP-dependent SERCA, and is released back into the cytosol through the IP3R or RyR calcium channels. Calcium can also flow from the ER to the mitochondria. During pregnancy, placental hormones bind to PrlR, inducing a signal cascade that results in up regulation of several genes to increase insulin production and secretion. These include Lrrc55 and SERCA to maintain [Ca^2+^]_ER_, MCU to maintain [Ca^2+^]_m_, and INS1/2 to increase insulin expression. VDAC, voltage-dependent anion channels; MCU, mitochondrial Ca^2+^ uniporter; PRLR, prolactin receptor; Prl/PL, prolactin/placental lactogen. The dashed line arrow indicates that influx of calcium induces insulin secretion. Created with BioRender.com.

The electrically excitable pancreatic β-cells utilize the controlled flux of a few key ions, all of which coupled to calcium flux, to precisely regulate insulin release in response to high levels of blood glucose (**Figure 1**).

Glucose enters β-cells *via* the facilitated glucose transporter GLUT2 in rodents and GLUT1 and GLUT3 in humans. Upon phosphorylation by glucokinase, glucose-6-phosphate is metabolized by glycolysis and the TCA cycle to generate ATP. The increase in the ATP/ADP ratio results in closure of the K_ATP_ channels in the plasma membrane and a burst of action potentials, which leads to opening of the voltage-dependent Ca^2+^ channels (VDCC), allowing Ca^2+^ influx and an increase in [Ca^2+^]_c_, especially in submembrane areas near the Ca^2+^ channels. At moderate glucose concentration (7-15mM), the intermittent opening of VDCC causes [Ca^2+^]_c_ oscillation while at high glucose concentration (>20mM), the continued opening of VDCC causes a sustained increase in [Ca^2+^]_c_ (11). VDCC is not distributed uniformly throughout the plasma membrane, generating microdomains with high [Ca^2+^] (64). These subdomains of high [Ca^2+^]_c_ are in close proximity to the voltage-gated Ca^2+^ channels and form hot spots for insulin granule to dock and fuse (65), a process that is facilitated by soluble N-ethylmaleimide-sensitive factor attachment protein receptors (SNAREs) and SNARE regulator proteins, such as syntaxin 1A (66), SNAP-25 (67–69) and synaptotagmins (70–73). Repolarization of the plasma membrane results from the rapid inactivation of VDCC and the opening of potassium channels (Kv2.1 voltage-dependent channels and large-conductance Ca^2+^-activated K^+^ channels, (BK) (11, 74, 75).

Limited data are available on pregnancy-induced changes in intracellular calcium dynamics. In a study on the effect of protein restriction on insulin secretion and intracellular calcium concentration, Marin et al. reported that in islets from control (not protein restricted) rats, on day-15 of pregnancy, glucose elicited a larger and earlier rise in [Ca^2+^]_c_ in comparison to β-cells from non-pregnant rats. Interestingly, while this larger rise in [Ca^2+^]_c_ did not result in a change in the total amount of Ca^2+^ influx during plasma membrane depolarization, it was accompanied by a more sustained and gradual insulin secretion profile. Islets from pregnant rats also expressed more SNAP-25, although its specific role on calcium dynamics was not explored (76). Vanzela et al. reported that islets from pregnant rats (15^th^ or 16^th^ day of pregnancy) had increased calcium oscillation and a higher level of expression of Ca_v_α1.2 and SERCA2a in comparison to non-pregnant rats (77). In a model of cafeteria-diet (Caf) induced obesity, islets from Caf-exposed rats exhibited a blunted glucose-induced calcium increase and insulin secretion, both of which were reversed by pregnancy. The Caf-induced reduction in Ca_v_α1.2 expression was also reversed by pregnancy. They concluded that pregnancy reversed the deleterious effects of Caf on islet function by restoring calcium handling, in part through the up regulation of Ca_v_α1.2 and SERCA2a expression. Expression of the SNARE protein, synaptotagmin 4 (Syt4), has also been shown to be up regulated in islets isolated from rats on day 15 of pregnancy and in prolactin-treated islets *in vitro* (70). Syt4 regulates Ca^2+^ sensitivity in β-cells and its expression is increased by ~8-fold during β-cell maturation, leading to increased GSIS in mature islets in comparison to neonatal islets in mice (78). Syt4 is expressed in insulin vesicles, Golgi, and the ER, and potentially regulates general Ca^2+^ signaling in the ER of β-cells (78). Syn4 is expressed in human β-cells and it regulates insulin secretion in the human β-cell line EndoC-βH1 (78). Taken together, these changes in expression and activities of Ca^2+^ channels, SNARE proteins, and glucose metabolism may contribute to the changes in Ca^2+^ influx and insulin secretion in pregnancy.

## ER Calcium in β-Cells

ER calcium concentrations are mainly determined by the sarco/endoplasmic reticulum Ca^2+^-ATPase (SERCA), which actively pumps calcium into the ER from the cytosol, as well as two receptor-type membrane proteins, the ryanodine receptor (RyR) and the Inositol Triphosphate Receptor (IP3R) (79–81), both of which releases calcium from the ER. Blocking IP3R, RyR, and combinations thereof, has been shown to rescue β-cells from ER calcium depletion caused by SERCA inhibitor (82). Luciani et al. found that in the mouse β-cell line MIN6, blocking IP3R appeared to have a more pronounced effect on β-cell survival than blocking RyR (82). Interestingly, Hara et al. found that in conditions associated with β-cell death, such as ER stress, oxidative stress, exposure to palmitate, chronic high glucose, and overexpression of mutated insulin, there were decreases in ER calcium levels (in INS-1 cells) and SERCA expression (in INS-1, human and mouse islets) (83). They hypothesize that genetic and environmental stressors cause β-cell stress, leading to a reduction in SERCA2b and an increase in IP3R expression, resulting in a reduction in ER calcium and an increase in [Ca^2+^]_c_ leading to β-cell death. How ER stress causes a reduction in SERCA2b expression is currently unknown.

The increased insulin demand seen during pregnancy and the associated increase in protein synthesis can activate the unfolded protein response (UPR), which if unresolved, can lead to ER stress. Indeed, while we did not observe an increase in the number of apoptotic β-cells during mouse pregnancy (15), we found that expression of IRE1α and CHOP, components of the UPR pathways, are up regulated in mouse islets during early pregnancy (25). Anhe et al. reported that on d19 of pregnancy, SERCA2 expression was upregulated in rat islets. This increase may contribute to the increase in GSIS observed during pregnancy, as inhibition of SERCA2 activity by thapsigargin led to a reduction in 1^st^ phase insulin secretion in isolated rat islets (84). Activation of prolactin receptor may also contribute to these pregnancy-associated changes, as levels of prolactin and its related hormones, placental lactogens, are high throughout pregnancy, and treatment of the rat islet cell line, RINm5F, with prolactin recapitulated the increase in SERCA2 expression observed during pregnancy (84). Interestingly, prolactin has been shown to up regulate SERCA2b expression, increase [Ca^2+^]_ER_, and stimulate cell proliferation in prostate cells. Conversely, SERCA2b knockdown reduced both [Ca^2+^]_ER_ and cell proliferation (85). Whether primary β-cells would have a similar response to prolactin in terms of increasing [Ca^2+^]_ER_ has not been examined.

Protein kinase R (PKR)-like endoplasmic reticulum kinase (PERK) (EIF2AK3) is an eIF2α kinase in the ER membrane, known to regulate β-cell development, function, and ER stress response (13, 86, 87). PERK inhibition lowered the glucose-induced rise in intracellular calcium and blunted GSIS in INS-1 cells as well as in rat and human islets (88). PERK regulates intracellular calcium levels by at least two mechanisms: first, it controls calcium influx into the cytosol by regulating store-operated Ca^2+^ channel (SOCC) activity (89); second, it stimulates SERCA-mediated calcium reuptake into the ER after [Ca^2+^]_ER_ depletion or release *via* a calcineurin dependent pathway (88). During pregnancy, expression of PERK in the islets rises near the end of gestation (day 19 in mice) and peaks on day 1 of lactation, but promptly drops below pre-pregnancy level by day 2 of lactation (90). How and whether this increase in PERK expression contributes to pregnancy-associated changes in calcium dynamics in β-cells remains to be determined.

## Mitochondrial Calcium Dynamics in β-Cells

Calcium influx into the mitochondria is important for facilitating GSIS from β-cells. Under basal condition, intramitochondrial calcium ([Ca^2+^]_m_) is low, comparable to that of [Ca^2+^]_c_ (<100nM) (91). Calcium is transported into mitochondria through voltage-dependent anion channels (VDACs) in the outer mitochondrial membrane and the mitochondrial Ca^2+^ uniporter (MCU) (92) in the inner mitochondrial membrane, while calcium exits the mitochondria through the Na^+^/Ca^2+^ exchanger (NCLX) (93). Endoplasmic reticulum is another source of Ca^2+^ for mitochondria, and tethering molecules such as GRP75 and mitofusins are present in microdomains between ER and mitochondria to facilitate calcium flow from ER (which has basal calcium concentration of ~5mM) into the mitochondria (94, 95).

In mouse β-cells, Tarasov et al. demonstrated that [Ca^2+^]_m_ follows the slow but not fast changes in [Ca^2+^]_c_, and [Ca^2+^]_m_ is highly sensitive to calcium oscillation (91). At low glucose concentration, the small spikes in [Ca^2+^]_c_ is not transmitted to the [Ca^2+^]_m_ but as glucose levels rises, the increase in [Ca^2+^]_c_ oscillation leads to an increase in [Ca^2+^]_m._ The rise in intramitochondrial calcium activates mitochondrial dehydrogenases and further stimulates ATP production, resulting in a biphasic phase increase in [ATP/ADP]_c_. Tarasov et al. speculated that the second phase of ATP production may be involved in mobilization of the reserve pool of insulin granule (91).

While changes in [Ca^2+^]_m_ have not been directly determined in β-cells during pregnancy, it is tempting to speculate that the subtle changes in cytosolic calcium oscillation discussed above may influence [Ca^2+^]_m_, and explain in part the increase in insulin secretion observed during pregnancy. Expression of MCU increased throughout gestation in human placenta (96). Whether a similar change is present in pancreatic islets and the functional consequence to mitochondrial calcium handling is unknown.

## Calcium and Gestational Diabetes

Gestational diabetes mellitus (GDM) is defined as diabetes diagnosed for the first time during pregnancy (97). While estimates differ depending on the populations studied, approximately 3-20% of pregnancies are complicated by GDM (97, 98). GDM is associated with a higher risk of maternal and neonatal adverse outcomes, such as pre-eclampsia, macrosomia, stillbirth, and neonatal hypoglycemia (99). Women who have developed GDM are at high risk of developing GDM in subsequent pregnancies as well as progressing to type 2 diabetes. Additionally, exposure of the offspring to hyperglycemia *in utero* significantly increases their risk of developing Type 2 diabetes later in life (14, 100, 101).

Many human studies have examined the relationship between serum calcium and vitamin D levels and the risk of diabetes (102–104). A large epidemiological study in >3400 US women found that periconceptional calcium intake was inversely associated with the risk of developing GDM (105). They hypothesized that the relationship between calcium intake and GDM risk may lie in the positive association between intracellular calcium ([Ca^2+^]i) and insulin secretion in β-cells; they also observed a U-shaped relationship between [Ca^2+^]i and insulin sensitivity in vascular smooth muscle and adipocytes (106). Calcium sensing receptor (CaSR) may potentially link serum calcium levels to β-cell function. CaSR is expressed in β-cells and it contributes to β-cell adhesion, coupling, and communication. CaSR has also been shown to inhibit basal and GSIS in human islets (107). Transgenic mice with gain-of-function mutation of CaSR have reduced islet mass and β-cell proliferation, as well as hypoinsulinemia and hyperglycemia (108). In human pregnancies complicated by GDM, expression of CaSR was found to be significantly reduced in the placenta, which may have contributed to the hypocalcemia observed in 16% of the newborns (109). Whether CaSR expression was also altered in islets of women with GDM could not be determined due to the inaccessibility of pancreatic tissue for analysis.

In support of the link between calcium dynamics and GDM risk, Goldstein et al. used an informatics-based approach to determine the association between GDM and/or type 2 diabetes, disease-associated SNPs, and the effects of a list of 129 active drugs in 9960 patients. They found that the use of a calcium channel blocker (CCB) such as nifedipine was associated with a reduction in serum glucose during glucose tolerance tests, and there was a strong association between genes targeted by CCBs and GDM risk (110). Mechanistically, treatment of mouse islets with CCB increased basal insulin secretion and reduced glucagon secretion (111) while blocking calcium entry was shown to protect β-cells and human islets against ER stress and apoptosis (112–114).

The level of circulating vitamin D (25-hydroxyvitamin D [25(OH)D]) during pregnancy has also been shown to inversely correlate with GDM risk (103, 115). In a meta-analysis, Wei et al. reported that low levels of circulating vitamin D increase the risk of GDM by 1.38-fold (116). Vitamin D levels may contribute to GDM *via* its putative effect on both insulin sensitivity and insulin secretion (117). Non-genomic signaling through vitamin D receptor has been shown to augment GSIS by increasing intracellular Ca^2+^ concentration, which was blocked by the CCB nitrendipine (118). Norman et al. found that pancreas from vitamin D deficient rats showed a 48% reduction in GSIS in an ex-vivo perfusion experiment (119). Moreover, in a study of 126 healthy human subjects, serum vitamin D levels were found to negatively correlate with 1^st^ and 2^nd^ phase insulin release during a hyperglycemic clamp, and an effect on β-cell function remains after correction for insulin sensitivity index (120).

## Conclusion

In this review, we discussed the current knowledge of calcium dynamics in pancreatic islets during pregnancy. The hallmark of β-cell adaptation to pregnancy is an increase in insulin secretion, a process that is tightly regulated by intracellular calcium dynamics. Hormones and small molecules such as estrogen, corticotropin releasing hormone, serotonin, HGF, and placental lactogens potentially enhance insulin secretion and β-cell proliferation *via* regulating calcium dynamics. Furthermore, up regulation of GLUT2 and glucokinase during pregnancy allows more efficient glucose metabolism. Coupled with the increased expression of L-type Ca^2+^ channel, these changes allow β-cells to secrete insulin more efficiently. The finding that calcium channel blockers improve glycemia is very exciting and encouraging, as it points to abnormal intracellular calcium dynamics as a potential contributing factor to GDM but it also provides a safe and effective treatment for those with GDM. Currently, our understanding of how intracellular calcium dynamics changes as part of β-cell adaptation to the increased insulin demand of pregnancy is very limited and fragmented. A thorough understanding of this field would allow for design of more targeted therapy for GDM and prevent the vicious cycle of GDM begetting more GDM.

## Author Contributions

MP and CH wrote the manuscript. Both authors contributed to the article and approved the submitted version.

## Funding

This work was supported by funds from Natural Sciences and Engineering Research Council of Canada (RGPIN-2020-05247) to CH.

## Conflict of Interest

The authors declare that the research was conducted in the absence of any commercial or financial relationships that could be construed as a potential conflict of interest.

## Publisher’s Note

All claims expressed in this article are solely those of the authors and do not necessarily represent those of their affiliated organizations, or those of the publisher, the editors and the reviewers. Any product that may be evaluated in this article, or claim that may be made by its manufacturer, is not guaranteed or endorsed by the publisher.
